# NIH support of mobile, imaging, pervasive sensing, social media and location tracking (MISST) research: laying the foundation to examine research ethics in the digital age

**DOI:** 10.1038/s41746-017-0001-5

**Published:** 2018-01-15

**Authors:** Sarah Dunseath, Nadir Weibel, Cinnamon S. Bloss, Camille Nebeker

**Affiliations:** 1 School of Medicine, University of California San Diego, La Jolla, CA USA; 2 Center for Wireless and Population Health Systems, Qualcomm Institute, University of California San Diego, La Jolla, CA USA; 3 Department of Computer Science and Engineering, University of California San Diego, La Jolla, CA USA; 4 Department of Psychiatry, School of Medicine, University of California San Diego, La Jolla, CA USA; 5 Department of Family Medicine and Public Health, School of Medicine, University of California San Diego, 9500 Gilman Drive, MC-0811, La Jolla, CA USA

**Keywords:** Funding, Translational research, Ethics

## Abstract

Mobile Imaging, pervasive Sensing, Social media and location Tracking (MISST) tools used in research are raising new ethical challenges for scientists and the Institutional Review Boards (IRBs) charged with protecting human participants. Yet, little guidance exists to inform the ethical design and the IRB’s regulatory review of MISST research. MISST tools/methods produce personal health data that is voluminous and granular and, which may not be subject to policies like the Health Information Portability and Accessibility Act (HIPAA). The NIH Research Portfolio Online Reporting Tools (RePORTER) database was used to identify the number, nature and scope of MISST-related studies supported by the NIH at three time points: 2005, 2010 and 2015. The goal was to: 1-examine the extent to which the NIH is supporting this research and, 2-identify how these tools are being used in research. The number of funded MISST research projects increased 384% from 2005 to 2015. Results revealed that while funding of MISST research is growing, it only represented about 1% of the total NIH budget in 2015. However, the number of institutes, agencies, and centers supporting MISST research increased by roughly 50%. Additionally, the scope of MISST research is diverse ranging from use of social media to track disease transmission to personalized interventions delivered through mobile health applications. Given that MISST research represents about 1% of the NIH budget and is on an increasing upward trajectory, support for research that can inform the ethical, legal and social issues associated with this research is critical.

## Funding: support for mobile technologies on the rise

The number of US government-backed biomedical research projects that involved mobile and digital technologies rose 384% from 2005 to 2015. Camille Nebeker and colleagues from the University of California, San Diego, USA, tabulated how much grant money the US National Institutes of Health was allocating to research projects in which these new kinds of imaging, sensing and tracking tools played an integral role. They found that the agency spent $47 million on 134 relevant projects in 2005; $137 million for 338 projects in 2010; and $293 million for 649 projects in 2015. Although these studies in 2015 represented only about 1% of the agency’s total budget, the authors conclude that the growing popularity of the technologies necessitates giving more consideration to the ethical, legal and social issues associated with their use with human research subjects.

## Introduction

Mobile Imaging, pervasive Sensing, Social media and location Tracking (MISST) technologies including but not limited to mobile applications or “apps”, fitness tracking devices and social networking platforms are becoming increasingly prevalent in present society. At the same time as these devices are being used by individuals as sources of entertainment, organization, and socialization, they are also being used by scientists to conduct research in areas ranging from disease transmission,^1^ to substance abuse^2^ and weight loss.^3^ These digital and pervasive sensing technologies are becoming valuable tools for many scientists; however, researchers and the institutional review boards (IRBs) charged with protecting human research participants are struggling to understand how to best inform prospective participants, as well as manage the collection, analysis and sharing of data acquired through these technologies.

The novelty combined with a lack of experience and technical expertize makes calculating the probability and magnitude of potential harms and benefits to research participants difficult. Likewise, determining appropriate risk management strategies are also unclear. These new challenges combined with static federal regulations to guide human research protections and ethical principles^4^ developed when research was conducted using more traditional methodologies, may contribute to longer than typical IRB review periods-potentially hindering the progress of health research.^5^ Given the incredible possibilities of these mobile and digital technologies in the context of biomedical research, it is critical to reflect on how to conduct ethical and responsible research as we design and roll out MISST studies. We are now embarking on a period where research is designed to enroll 10 thousand to 1 million participants (e.g., National Institutes of Health's *All of Us* Research Program, Google’s Baseline, Apple’s ResearchKit) and, in order to guide the critical and timely development of ethical practices, we need to better understand the MISST ecosystem and the extent to which this research is occurring.

The work presented here characterizes and quantifies MISST research funded by the National Institutes of Health (NIH) at three time points: 2005, 2010, and 2015. To achieve this goal, we mined the NIH Research Portfolio Online Reporting Tools (RePORTER) database to extract information that aligned with the use of MISST technology. Previous studies have used the RePORTER tool to foster communication between researchers,^6^ examine allocation of funding for specific areas of research,^7^ examine gender disparities related to research funding grants,^8^ and to identify and categorize funded projects related to social issues,^9^ but it has not previously been used to examine the MISST landscape. These previous studies and use of the RePORTER search tool, specifically the keyword search used in Coulter et al. in 2014,^9^ inspired the approach applied to this study.

## Results

Analysis of the data revealed an overall increase in the quantity and scope of MISST research from 2005 to 2015. We observed both an increase in the quantity and funding of MISST research, as well as an increase in the proportion of the NIH budget allocated to MISST research, the number of institutes, agencies and centers supporting MISST, and the number of institutions conducting MISST research. In the remainder of this section we detail this increasing support for MISST research.

### MISST overall funding: awards and topics

In the ten-year period between 2005 and 2015, the number of awards allocated to MISST research by the institutes, agencies, and centers included on the NIH RePORTER increased 384% from 134 awards in 2005 to 649 awards in 2015. In 2005, research using MISST technologies comprised approximately 0.17% of the total NIH budget, receiving a total of approximately $47 million in funding. In 2010, MISST research represented approximately 0.45% of the total NIH budget (2005 and 2010 funding information determined from the “NIH Budget Mechanism Detail FY 2001-2014” on NIH RePORTER) receiving a total of approximately $137 million, and in 2015 it doubled to approximately 1% of the total budget (2015 funding information determined from supplementary tables from “Budget Request by Institute and Center” provided by the NIH Office of the Budget), receiving more than $293 million in funding.

When looking at the kind of MISST studies that were awarded, it is interesting to note how trends include the increase in use of mobile device/applications from 19.0% of all MISST research in 2010 to 47.6% in 2015. In addition, social media, which was not an integral component of any project receiving federal funding in 2005, comprised 5.2% of MISST research in 2010 and 14.9% in 2015. Finally, the number of studies using GIS/GPS technology, although it did not increase as rapidly as other MISST technology, also grew 36% from 2005 to 2015. In 2005, GIS/GPS was the most commonly used MISST technology, and represented 51.0% of all NIH-supported MISST research. By 2015, GIS/GPS was used in only 13.3% of all NIH-supported MISST research, reflecting the surge in use of other MISST technologies such as mobile devices/applications and social media (Table 1).

**Table 1:** Keywords and phrases used to search the NIH RePORTER database

Mobile	Imaging	Pervasive sensing	Social media	Location tracking
Cell phone	Camera based	Accelerometer	Facebook	Geographic information systems
mHealth	Camera-based	Actigraph	Instagram	GIS
Mobile app	Camera sensor	FitBit	Instant messenger	Global positioning system
Mobile application	Outward facing camera	Fitness tracker	MySpace	GPS
Mobile based device	Outwardly facing camera	Pedometer	Skype	Location tracking
Mobile device	Photovoice	SenseCam	Social media	
Mobile health	Wearable camera	Wearable sensor	Twitter	
Mobile technologies		Wearable sensors	YouTube	
Mobile technology		Wearable technology		
Mobile-based device		Wearable technologies		
Palm pilot				
Personal digital Assistant				
Smart phone				

### Changes in funding sources of MISST research

**Fig. 1:**
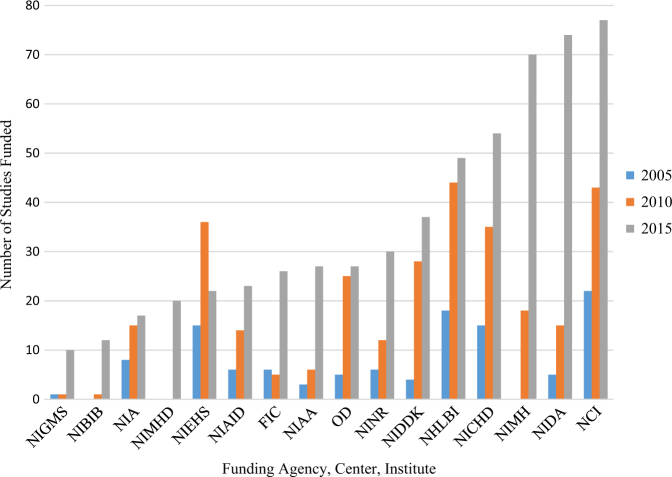
Number of MISST studies funded per institute in 2005, 2010, and 2015 of the institutes funding at least 10 projects at any of the three time points

In addition to cumulative increases in the number and amount of funding allocated to MISST research, the number of institutes, agencies, and centers supporting this research grew from 23 in 2005 to 34 in 2015. To contextualize this increase, the number of institutes, agencies, and centers were relatively static, reflecting an increased rate of support exceeding the rate of growth the NIH and its affiliated agencies and centers. The number of funded studies per institute/agency/center also increased from a median of three studies per funding organization to eight studies per funding organization (Fig. 1).

**Fig. 2:**
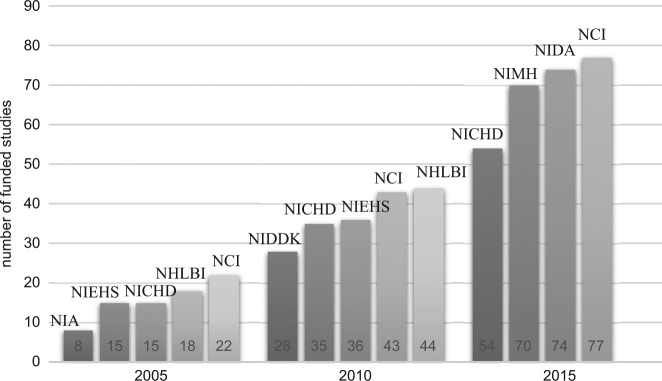
Top agencies, institutes, and centers included in the NIH RePORTER database funding the greatest number of MISST projects

We also looked at the institutes funding the greatest number of MISST projects at each time point. Two institutes were early supporters including: the National Cancer Institute (NCI), which funded 22 projects in 2005 and 77 projects in 2015, and the National Heart, Lung, and Blood Institute (NHLBI), which funded 44 projects in 2010 (Fig. 2).

**Fig. 3:**
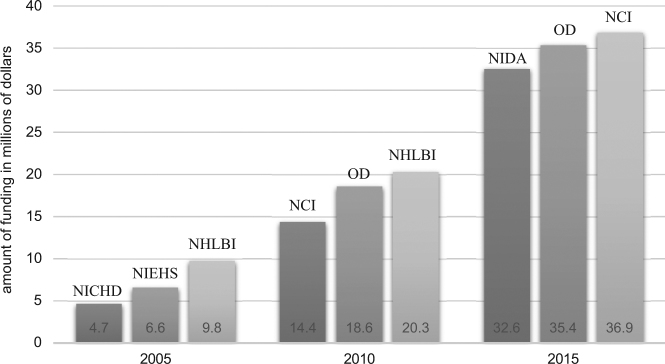
Top three agencies, institutes, and centers included in the NIH RePORTER database funding the greatest amount of MISST projects in millions of dollars

Analogously, the institutes allocating the greatest amount of funding for MISST research were again the NHLBI, which granted $9.8 million in 2005 and $20.3 million in funding in 2010, and the NCI, which granted $36.9 million in funding in 2015 (Fig. 3). However, it is also key to note how the National Institute of Mental Health (NIMH), which funded no MISST research in 2005, funded the third greatest number of MISST research projects in 2015, culminating in 77 projects and $24.8 million in funding.

### Scope of MISST research

As the amount of MISST research has increased, the scope or variety of research has changed. For example, the NCI funded 22 MISST projects in 2005. Out of these 22 MISST projects 40.9% were related to physical activity and/or obesity, 40.9% examined contextual factors, such as how location or demographic affected participants’ actions, 13.6% were related to exposure to pollutants or chemicals, 4.5% were related to disease/condition management, and 4.5% used MISST technologies in community-based interventions. Note that projects were not limited to one theme category, so a project could be classified as being related to physical activity and/or obesity and to exposure to pollutants or chemicals or to any other possible combination of multiple themes.

In 2010, NCI funded 43 MISST projects. Of these, 58.1% were related to physical activity and/or obesity, 32.6% were related to contextual factors, 18.6% were related to alcohol/drugs/tobacco use, 14.0% related to exposure to pollutants or chemicals, 4.7% were related to researcher training and data management, and 2.3% were related to disease transmission.

In 2015, themes expanded to include projects related to alcohol/drug/tobacco use, disease transmission, and researcher training and data management. Of the 77 MISST studies receiving NCI funding in 2015, 35.1% were related to physical activity and/or obesity, 31.2% to alcohol/drugs/tobacco use, 24.7% examined contextual factors, 15.6% related to disease/condition management, 11.7% related to researcher training and data management, 1.3% examined disease transmission, and another 1.3% related to exposure to pollutants/chemicals.

### Recipients of MISST research funding

In addition to the increasing amount and diversity of MISST research, the number of institutions conducting MISST research also increased. In 2005, 47 organizations received funding for MISST research and in 2010, 170 received support. By 2015 this number increased to 267, reflecting an increase of almost 470% since 2005. Additionally, the volume of MISST research generated by these institutes also increased and several institutions have emerged as being early pioneers. For example, in 2005 University of California, San Diego received funding for five MISST studies conducted by four different principal investigators (PIs), more than any other institution at that time. In 2015, the University of California, San Francisco received the most awards with twenty-two projects launched by seventeen different PIs. Although the introduction of MISST research expanded vastly over these time points, it is interesting to note how the University of California faculty have distinguished themselves in the use of emerging technologies in human-based research having received nearly $33 million in funding for a total of 68 projects in 2015.

## Discussion

This paper identified changes and trends in MISST research at three time points: 2005, 2010, and 2015. Interpretation of results requires careful consideration of the developments made to MISST technologies across these time points. For example, a researcher in 2005 seeking to examine patterns in disease transmission may have had to rely on handheld Global Positioning Systems (GPS) to obtain data and Geographic Information Systems (GIS) for data analysis. In 2015, a researcher with the same focus could employ new MISST technologies including more advanced mobile devices and applications or they could analyze Twitter content to examine locations and sources of disease transmission. It is particularly important to consider the advancements made in mobile devices and social media across these time points. In 2005, smartphones like the Apple iPhone did not yet exist and social media was in its infancy, being largely limited to MySpace, which was only two years old, and Facebook, a common social media platform used worldwide nowadays had launched only one year before. The thirteen-fold increase in the number of studies using mobile devices and applications from 2005 to 2015 might be largely explained by the increased capabilities of mobile devices and increased availability of these technologies due to decreased cost. The increase may also be partially accounted for by the delay in time between when a technology is first released to consumers and the time when it is incorporated by researchers into research proposals.

Along with the overall technology development, the surge in social media as a research tool may also be explained by the large increase of data that has been made available through these outlets, making them more valuable resources for researchers. In fact, as revealed by the Pew Research center, only 7% of American adults had social media accounts in 2005, but by 2015 that number increased to 65%.^10^ More specifically, Facebook increased from six million users in December 2005 to having over one billion users accessing the website daily in 2015 (Data collected from Facebook’s website under “company info”). Other MISST technologies have also surged in terms of their daily use in the general population. The capabilities of mobile phones have expanded significantly since 2005 and have made other electronics, such as cameras, pedometers, and location trackers nearly obsolete. As a result, most people with a modern smartphone are also carrying a camera, GPS, accelerometer and other technologies with them daily, providing researchers with novel opportunities for data collection.

### Limitations

This study is limited, to some extent, by the selection of keywords used to identify MISST-related research. Likewise, limiting the study to MISST does not capture the growth of research using technologies like virtual reality, robotics, 3-D printing and artificial intelligence. The database utilized for this study included only research funded by the NIH and its affiliated agencies and centers in the United States. Future studies could include other federal agencies, such as the National Science Foundation, and private foundations that are supporting MISST research.

## Conclusions

In this paper, we examined to what extent MISST research has been supported by the NIH over a ten-year period at three time points. We quantified awards and dollar amounts for this type of research and described the domains of research in which MISST is being used most frequently. What emerged is a clear increase in the use of MISST tools/methods applied to biomedical and behavioral health research. Specifically, we learned that MISST research has increased 384% in little more than a decade and has nearly doubled in the past 5 years yet, still reflects only about 1% of NIH expenditures. There is every reason to believe that how research is conducted in the 21^st^ century will continue to leverage emerging technologies to observe and intervene in human behavior with a goal of improving health.

By knowing who is supporting MISST research and where it is occurring, we are now able to examine the extent to which ethical guidelines have developed concurrently to assist in the responsible design (researchers) and ethical review (IRBs) of research using MISST. Recent studies analyzing the current scientific landscape around MISST repeatedly found that researchers and IRBs^11^ are struggling with understanding how to handle the increasing availability of these data from an ethical point of view.^12,13^ Even regulatory bodies like the IRB face difficulties, as shown in our prior work where we learned how risk assessment was often inconsistent and how policies to guide decision-making were non extant, leading to delays in grantees initiating their NIH supported research.^14^ We believe that future steps for advancing the field of MISST research must include examining how universities are responding to new research methods and whether guidance is being developed concurrently to inform the ethical design and application of federal regulations to protect research participants. As MISST research continues to increase and be used in novel ways, it is equally important that both researchers and regulatory bodies stay informed in order to provide appropriate protections of research participants. We call here for future research to examine whether and how institutions are applying federal regulations and evaluating the ethical, legal and social issues of MISST research with a focus on informed consent, risks assessment and data management challenges. Additionally, in order to promote development and discussion of new ethical frameworks that support this increase in MISST research, conversations regarding the ethical dimensions of new research tools and methods must be encouraged within institutions, professional organizations and at national venues where stakeholders come together. As we enter this age of digital medicine and mobile health and begin to implement major nationwide studies like the NIH's Precision Medicine Initiative *All of Us* Research Program, it is imperative that technologists, ethicists, regulators, researchers, privacy experts and research participants be included in shaping and iterating the ethical frameworks intended to inform potential participants and protect participant privacy. Likewise, standards for securing and sharing of research data must be developed as much of the personal health data collected by research grade and commercial devices and apps are not regulated if not part of a person’s medical record. We strongly believe that hand in hand with the increased funding of studies utilizing MISST technologies we need to increase awareness of MISST benefits and risks through the education of stakeholders. This will allow us, as a scientific community, to collectively make more informed decisions about what constitutes ethical MISST research practices, and best support the incredible advances that this technology is enabling.

## Methods

Data analyzed for this study were accessed using the NIH RePORTER’s “text search” feature. Since this research did not involve human research participants, IRB review was not sought. Our NIH RePORTER search targeted research that received funding from the NIH or its associated agencies and centers in the fiscal years 2005, 2010, and 2015. We chose 2005 as the start date because it was recent enough to include analogous or precursor technologies to 2015 MISST technologies, yet also long enough ago to reflect the evolution and trends in the MISST technology landscape.

The web-based query form was used to identify potentially relevant projects and their funding agency, institute, or center using 43 keywords and phrases. During our data gathering, all phrases and keyword were searched using quotations to eliminate irrelevant search results. To identify these 43 keywords and phrases we first identified MISST tools or methods of data collection. For example, we identified mobile apps and listed words like “Instagram” and “Twitter” yet, these terms popular in 2015, had not yet been introduced in 2005. To reduce a bias towards 2015-relevant technologies, we identified terms to equate with equivalent technologies from 2005. In the context of mobile devices for example, “personal digital assistant” or “palm pilot” were relevant in 2005 and we deemed those to be analogous to 2015 studies using present day mobile applications. Similarly, “pedometer” could be used as the 2005-equivalent for “Fitbit” or “fitness tracker.” Creating time-relevant terms for social media proved to be more challenging given the relative dearth of social media in 2005 compared to the robust number of social media platforms in 2015. The terms “social media,” “MySpace,” “Instant Messenger,” “YouTube,” “Facebook,” and “Skype” were used to reflect common social media platforms across these time points. Table 2 lists the final keywords by MISST category.

**Table 2:** Exclusion process for awards funded by the NIH, agencies and centers listed on NIH RePORTER in 2005, 2010, and 2015

Year	2005	2010	2015
Funding awards from the NIH, agencies and centers on the NIH RePORTER	62,102	75,533	61,970
Awards excluded because “MISST” does not appear in title	61,945	75,159	61,206
Remaining potentially relevant funding MISST research awards	157	374	764
Excluded: use of MISST technology not integral to study	4	12	69
Excluded: acronym in keyword search has alternative meaning unrelated to MISST	6	15	19
Excluded: MISST use in medical device development only	0	2	9
Excluded: MISST keyword used in analogy only	5	6	6
Excluded: keyword “mHealth” did not refer to MISST	0	0	5
Excluded: MISST use in nonhuman research	8	1	5
Total MISST funding awards	134	338	649

In 2005, 125 MISST research projects received funding from the NIH or an affiliated agency or center. However, in order to track the funding awarded from each institute-each award was classified as an individual project. Therefore, a project receiving either multiple awards from the same institute or multiple awards from different institutes was counted as “multiple.” For 2005, unique projects resulting in a total of 134 awarded projects - meaning of the 125 projects, 9 received funding from multiple sources). For example, a location tracking study from the University of Alaska Anchorage received funding from the NIH Office of the Director and from the National Institute of on Alcohol Abuse and Alcoholism. In order to accurately sum the cumulative financial support given by each institute per year, projects such as these were classified as two studies.^15^ In 2010, 293 MISST research projects received NIH funding, and of those, 45 received multiple sources of support which then totaled 338 unique funding awards counted for 2010. In 2015, 595 MISST research projects received NIH funding and 649 funding awards were granted-54 of the 595 received multiple sources of support. While conjecture at this point, that multiple institutes provided support for several single projects may mean that these institutes have a shared value for advancing studies that utilize novel tools and methods.

### Data exclusion

Projects yielded through multiple keyword searches were accounted for and duplicate studies, defined as studies generated through multiple keyword/phrase searches, were reconciled. Our goal was to examine the landscape of MISST research by identifying projects in which MISST technologies played an integral role. In order to maintain this focus and to prevent the inclusion of studies with minimal MISST involvement, only studies in which the MISST-related keywords/phrases appeared in the title or abstract were further analyzed. The validity of this approach was evidenced when looking at relevant studies in 2015, after these precautions were applied, nearly 10% of the potentially relevant studies were omitted due to the lack of MISST technologies playing a relevant role in their methodologies. This initial process identified 157 potentially relevant projects using MISST technology in 2005, 375 in 2010, and 764 in 2015 (Table 3).

**Table 3:** Total number of times each category of MISST technology was used in NIH funded research in 2005, 2010, and 2015

Technology	2005	2010	2015
	Total MISST Use (*n* = 147), (%)	Total MISST Use (*n* = 419), (%)	Total MISST Use (*n* = 767), (%)
Mobile	28 (19.0)	110 (26.2)	365 (47.6)
Imaging	4 (2.7)	25 (6.0)	19 (2.5)
Sensing	33 (22.5)	134 (31.9)	129 (16.8)
Social Media	0 (0.0)	22 (5.2)	114 (14.9)
Tracking	82 (55.8)	128 (87.0)	140 (18.2)

Please note that the total MISST device use for each year exceeds the number of funded studies because some projects utilized multiple MISST devices

After this first data extraction, we further manually analyzed the resulting projects to ensure validity. The lead author (SD) read each abstract to determine if it met any of the following six exclusion criteria and should therefore not be part of the results: (1) an acronym from the keyword search had alternative, non-MISST, meaning (e.g., GPS used as abbreviation for genome parsing suite) (2) a keyword/phrase was used in analogy only (e.g., comparing the navigation of surgical tool to having GPS); (3) a project was limited to nonhuman, preclinical research; (4) MISST technology was not integral to the study (e.g., participants were added to a Facebook page to enhance communication but not to aid in any sort of data collection); (5) a project was limited to medical device development and did not involve human research participants (e.g., enhancing surgical camera scopes); or (6) a project was limited to mobile health projects not using MISST methods or tools (e.g., use of mobile health care van as opposed to a mobile health cell phone application). This process of elimination resulted in our final list of 134 relevant MISST projects in 2005, 338 in 2010, and 649 in 2015, while 175 projects were excluded.

### Data coding

For each included project we further extracted the specific NIH funding institute, agency, or center, as well as the type(s) of MISST technology used and the total amount of funding received. During this process, we observed the increasing breadth of MISST research conducted between 2005 to 2015, and subsequently generated a list of recurrent themes of the topics studied and the technology utilized. Each abstract was then coded and organized using the following nine themes: (1) substance use, (2) disease transmission, (3) environmental or social contextual factors, (4) disease or condition management, (5) physical activity and/or obesity, (6) community-based intervention, (7) data management and/or research training, (8) analysis of social media posts, or (9) other. In order to ensure high internal validity, a second researcher in our team (RAK) reviewed a random sample of 10% of projects from each time point and coded them independently. There was 97% (68 out of the 70 study sample) agreement between the two reviewers. The two disputed studies were then discussed until the two reviewers reached 100% agreement.

It is important to note that one project initially included in our analysis had received $239 million in funding from the National Library of Medicine. After careful consideration, this project was excluded from our data given that the extremely high amount of funding was a significant outlier when compared to all other funding awarded (e.g., the next highest award was in 2010 and received $6.9 million). Had this project been included in analysis, it alone would have accounted for almost two thirds of the total MISST funding from all institutes, agencies, and centers in 2010 while the other 338 projects would have comprised only a third of the total funding for that year.

### Data availability

Data collected from NIH Research Portfolio Online Reporting Tools.
